# Odorant-Binding Proteins as Sensing Elements for Odour Monitoring

**DOI:** 10.3390/s18103248

**Published:** 2018-09-27

**Authors:** Paolo Pelosi, Jiao Zhu, Wolfgang Knoll

**Affiliations:** Austrian Institute of Technology GmbH, Biosensor Technologies, Konrad-Lorenzstraße, 24, 3430 Tulln, Austria; jiao.zhu@ait.ac.at (J.Z.); Wolfgang.Knoll@ait.ac.at (W.K.)

**Keywords:** odorant-binding proteins, chemosensory proteins, Niemann-Pick C2 proteins, site-directed mutagenesis, biosensors

## Abstract

Odour perception has been the object of fast growing research interest in the last three decades. Parallel to the study of the corresponding biological systems, attempts are being made to model the olfactory system with electronic devices. Such projects range from the fabrication of individual sensors, tuned to specific chemicals of interest, to the design of multipurpose smell detectors using arrays of sensors assembled in a sort of artificial nose. Recently, proteins have attracted increasing interest as sensing elements. In particular, soluble olfaction proteins, including odorant-binding proteins (OBPs) of vertebrates and insects, chemosensory proteins (CSPs) and Niemann-Pick type C2 (NPC2) proteins possess interesting characteristics for their use in sensing devices for odours. In fact, thanks to their compact structure, their soluble nature and small size, they are extremely stable to high temperature, refractory to proteolysis and resistant to organic solvents. Moreover, thanks to the availability of many structures solved both as apo-proteins and in complexes with some ligands, it is feasible to design mutants by replacing residues in the binding sites with the aim of synthesising proteins with better selectivity and improved physical properties, as demonstrated in a number of cases.

## 1. Introduction

Modelling the human sense of smell with biomimetic electronic devices has been a several-decades dream, soon after biochemical research had been applied to olfaction with the discovery of the first proteins involved in odour detection [1,2,3]. The first attempts to design an electronic nose used poor specificity gas sensors, based on metal oxides [4] and conducting polymers [5,6]. During the last four decades, improvements have been made in the fabrication of sensors, signal transduction techniques and data processing [7,8,9,10], but we are still far from designing an instrument capable of discriminating and recognizing odours in a way similar to the human olfactory system.

There are two major difficulties still preventing the accomplishment of such goal: (1) our knowledge of odour coding in the human nose is still poor, and (2) suitable gas sensing elements with required sensitivity, stability and flexibility are still lacking. The need for information about the biological system is essential when designing an electronic nose, defined as an analytical instrument capable of discriminating odours using an approach similar to the one realized in our olfactory system. Aspects related to the olfactory code and strategies currently adopted to decipher the language of smell have been already discussed in a recent review [11]. Therefore, here we focus on the choice of sensing elements and suggest, on the basis of several experimental aspects, that proteins, in particular soluble binding proteins of olfactory systems, could qualify as suitable detecting elements for artificial gas and smell sensing.

Our first reason for preferring proteins is that, with the olfactory system being rather specific in recognising different chemical structures of volatile compounds, proteins are very likely the best candidates and probably the obligated choice as sensing elements with the required selectivity. There are however problems associated with the use of proteins in electronic systems, as they can be easily denatured or degraded, while needing a moist environment to keep their structure and consequentially their functionality. Despite such difficulties, proteins are now regarded as the best alternative to be incorporated into an artificial nose and successful results have been recently published [12,13,14,15,16,17,18,19].

It is worth emphasizing at this point that, although binding proteins appear as the most promising biosensing elements, sensitivity remains probably the main issue in artificial olfaction. Taking as a reference the human nose (which is a poorly performing system when compared to olfaction in insects [3] and also in other mammals), the most sophisticated devices are still orders of magnitudes far from the desired goal [11]. To fill such a gap we need new approaches, rather than more sophisticated amplifiers.

Being any volatile compound a potential odorant, in principle any protein with binding affinity to small hydrophobic chemicals could represent a suitable odour detector candidate. However, the first suggestions are provided by our biological system providing binding proteins which are suitable for technological applications. Our sense of smell utilises two classes of proteins that have been shown to bind smell molecules with good affinity and specificity: membrane-bound olfactory receptors (ORs) and soluble odorant-binding proteins (OBPs). While ORs would represent the first most obvious alternative, as they are responsible for detecting and discriminating different odorant molecules [20,21,22,23,24], their nature as membrane receptors and their related poor stability in harsh environmental conditions could severely limit their use in artificial devices. Moreover, overexpression of both vertebrate and insect ORs in heterologous systems proved difficult and affected by poor yields [25,26,27,28,29]. We therefore focus in this review on the use of OBPs and other soluble binding proteins as the most promising biosensing elements to be incorporated into electronic detectors.

## 2. Structure and Function of Odorant-Binding Proteins

The structural and functional characteristics of OBPs have been discussed in several recent reviews [30,31,32,33]. Here it will suffice to briefly summarize the main features of these soluble proteins with a specific focus on their potential use in biosensors and their suitability to be incorporated into electronic measuring instruments. We shall then describe in more detail the application of site-directed mutagenesis to modify the binding specificities and the physical properties of these proteins, reporting few examples where such strategy has been successfully adopted.

### 2.1. Soluble Proteins of Chemical Communication

There are at least four families of soluble binding proteins recognised to act as carriers for pheromones and odours in chemical communication. Two of them bear the same OBP name, despite being structurally different, with the OBPs of vertebrates [34,35,36,37,38], presenting the typical β-barrel folding of lipocalins [39,40] and the OBPs of insects made instead of α-helical segments [33,41,42]. So far the structures of only eight vertebrate OBPs have been solved [34,35,36,37,43,44,45], in contrast with more than 30 insect OBP structures. Proteins similar to insect OBPs and therefore named OBP-like, have been recently reported in some non-insect arthropods, namely chelicerata (spider, ticks and mites), being absent in insects and in crustacea [46,47,48,49,50]. Members of the third family are called Chemosensory Proteins (CSPs) because they were found to be associated with chemosensory organs [51,52,53]. However, this group of proteins includes members involved in a variety of functions, all likely related to binding and transport of small hydrophobic compounds [30]. The structure of CSPs is also made of α-helical domains, as in the case of insect OBPs, but their folding is quite distinct. Three-dimensional structures are available for only three members of this family [54,55,56,57].

**Figure 1:**
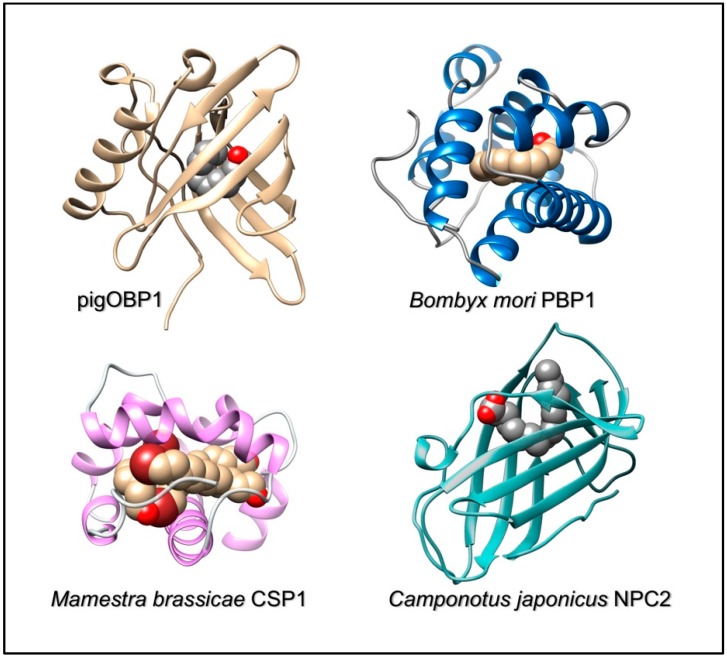
Three-dimensional structures of representative members of the four classes of soluble binding proteins so far described in chemical communication.

A fourth family of soluble binding proteins is now recognized as being involved in chemoreception. These proteins, named Niemann-Pick C2 (NPC2) after the discoverers of the related disease, have been known for a long time as carriers for cholesterol and other lipids in vertebrates [58,59]. Only recently, their large duplication and differentiation in arthropods has suggested a role in semiochemical detection and differentiation in insects and other arthropods [30,32,49,60,61,62]. The folding of these proteins, made of β-sheet segments, recalls that of lipocalins, being however well distinct from that of vertebrate OBPs. Figure 1 shows the structures of binding proteins representative of all the four classes described above.

All these binding proteins have been differentially adopted during evolution and occur in different subphyla, such as Chordata, Hexapoda, Chelicerata and others in a pattern still too unclear to establish relationships between these classes or with potential ancestors. We can also observe, within this perspective, that proteins of all four classes above described as odour and pheromone carriers, often perform other physiological functions both in vertebrates and invertebrates [30]. Table 1 summarizes the occurrence of these soluble proteins of chemical communication in different orders across evolution.

### 2.2. Stability of Proteins

All the families of odorant carrier proteins described above are exceptionally stable to thermal and solvent denaturation, as well as to proteolytic degradation. OBPs of both vertebrates and insects, as well as CSPs, have been shown to withstand extensive boiling without losing their binding characteristics when cooled down again to room temperature. They can be also completely unfolded by urea denaturation and reduction of disulphide bridges, and then fully renaturated in the original folding when brought back to standard conditions. Such protocol is actually adopted to solubilise these proteins when expressed in bacteria and produced as inclusion bodies [63,64,65].

Specific studies have addressed thermal unfolding of OBPs using infrared spectroscopy, spectrofluorimetry and circular dichroism [66,67,68,69]. Both vertebrate and insect OBPs are particularly stable to thermal denaturation, and their structure are only slightly affected up to temperatures of 70–75 °C. Moreover, the presence of ligands inside the binding pocket further increases the thermal stability of the protein [66].

### 2.3. Efficient Expression and Easy Purification

Proteins of all four classes have been successfully expressed in bacterial systems with yields of 20–50 mg/L. Depending on the protein, members of all four classes are often found in the soluble fraction, but occasionally they are expressed as insoluble bodies. In such cases, solubilization can be accomplished by denaturing (urea, guanidium chloride) and reducing (DTT) treatments, and then refolding by extensive dialysis. It has been found that in such cases proteins of all four classes refold in their native conformations during renaturation steps [63,64,65]. Most plasmids used to insert the genes into the bacterial cells produce the proteins inside the cytoplasm, but some of them send the mature protein into the periplasmic space. This method has the advantage of always producing the protein in its soluble form, associated to a simplified extraction procedure, not requiring breaking of the cells by sonication, but using a milder osmotic shock to release the periplasmic content [41,70]. However, yields in some cases are lower than with cytoplasmic expression.

Yeast expression has also been used with both mammalian [71] and insect [72] OBPs. Yields are quite good and the protein can be very easily purified from the external medium, being by far the most abundant component. However, being released into the large culture medium, they need to be concentrated before purification. One important aspect to bear in mind when considering yeast expression is related to post-translational modifications, such as glycosylation and phosphorylation, that have been occasionally found in native OBPs [45,73,74]. In most cases, when the proteins are used for sensor construction, such modifications are not needed and can even introduce problems, therefore bacterial expression may be preferred. However, it has been suggested that phosphorylation of OBPs could affect ligand-binding properties [75,76], therefore in the biological system such modification could be used as a way of regulating the selectivity of these binding proteins [75,76]. It is tempting to envisage an application of this mechanism in biosensing devices, where fast enzymatic processes, such as phosphorylation and dephosphorylation could be used to switch on and off a binding protein, thus providing a simple and efficient way of regenerating the biosensor. However more detailed studies on the properties of phosphorylated OBPs are needed.

Purification is usually accomplished by chromatographic steps, using different techniques. Thanks to the high expression level of these proteins and to the acidic nature of most of them, a first run of a crude bacterial extract on anion-exchange resins can afford the desired protein at a purity level of 80–90%. Final purification is generally achieved by a second anion-exchange chromatographic step or by gel filtration [63,64,65]. OBPs and other soluble proteins of chemical communication can also be purified in a single step by affinity chromatography on nickel columns if a His-tag segment had been added to the sequence. This tag has also been used to immobilise proteins on sensor surfaces in a regular and directional way [14].

### 2.4. Binding Assays

Measuring the affinity to volatile ligands, such as pheromones and odorants present in the environment is of basic importance in characterising these carrier proteins. When used in biosensing devices, it is important to verify that the responses of the artificial sensors reproduce the behaviour of the protein in solution.

Several techniques have been adopted to measure the affinity and the specificity of OBPs and other odorant carrier proteins to pheromones and general odours. A recent review provides an outlook of the advantages and drawbacks of each approach [65]. However, by far the most widely adopted method uses a fluorescent reporter, whose optical properties are markedly different when in an aqueous environment and when bound to a protein into a hydrophobic pocket [66]. This method is simple, fast and inexpensive in terms of the protein needed, thus allowing hundreds of measurements to be made in a couple of day, using as little as few milligrams of protein. Usually an external fluorescent probe is used as reporter: its emission spectrum generally undergoes a blue shift accompanied by a strong increase in fluorescence when the probe is bound inside the hydrophobic pocket of a protein. In this way, the amounts of fluorescent reporter bound and free can be evaluated without disturbing the equilibrium. Binding of other ligands can be evaluated in competition assays, where the amount of fluorescent probe is displaced by gradual addition of the second ligand. In some case it is possible to directly monitor the intrinsic fluorescence of the protein if a tryptophan residue is present in the binding pocket [65]. This approach, that however, presents some limitations for a general use, becomes more practical when using the proteins as biosensing elements. In fact, it avoids the use of an additional chemical, that must be continuously supplied to the system, as it is washed away at the end of each measurement. Most OBPs and CSPs of insects contain a tryptophan in their binding pocket, making this approach feasible without any modification. For other odour carrier proteins, however, a tryptophan can be easily added by site-directed mutagenesis. The main limitation of a tryptophan being used as a reporter is that its fluorescence is mainly affected by aromatic compounds, that can efficiently quench the signal by direct electron transfer. Moreover, the efficiency of tryptophan quenching cannot be related to affinity constants of different ligands, because such optical behaviour is strongly affected, even for aromatic compounds, by the place and orientation different ligands assume within the binding pocket. This is a major drawback when characterising the functionality of a protein, but does not represent a problem when using the protein as a detector for specific analytes.

While discussing the use of proteins to detect chemicals in biosensing devices, we can reverse the problem and look from a specular point of view. Can biosensors be used in monitoring the affinity of ligands to proteins, providing an alternative method to currently used fluorescent binding assays? There is a strong demand for label-free methodologies to measure binding constants of small ligands to proteins and certainly in the future biosensors might find applications in this field. At present, however, the available technology has not reached the level of sophistication required to ensure the required reproducibility in the fabrication of sensors [65].

### 2.5. Three-Dimensional Structures

Members of all four classes of binding proteins have had their structures solved either by X-ray crystallography or by NMR. By far the family with the largest number of known structures is that of insect OBPs. Since the folding of the *Bombyx mori* pheromone-binding protein (PBP) was first solved by crystallography [41] and soon after also by NMR [42,77], a large number of structures are available for this class of binding proteins. Table 2 provides a list of all the insect OBPs so far described at the structural level together with their accession numbers. Several members have been studied in complexes with ligands, thus providing a detailed picture on how the pheromone or the odorant interacts with residues inside the binding cavity. Such information represents a valuable data base when designing specific mutants for specific applications. Among the ligands, a couple of fluorescent reporters have been visualised inside the binding pockets, thus confirming at the structural level that such compounds do specifically bind OBPs and providing further experimental evidence to the fluorescent binding method [78,79].

The scaffolding of insect OBPs include six α-helical domains arranged in a compact structure further stabilised by three interlocked disulphide bridges [80,81]. Such structure has been very successful and is well conserved through proteins of different insect orders, from Orthoptera to Diptera, despite extensive variations in protein sequences. In fact, OBPs of insects can be as divergent as to share only 10% of their amino acid residues, but the six conserved cysteines with their disulphide bridges ensure a common architecture with a well-defined hydrophobic binding site. This is true for most insect OBPs, referred to as “classic OBPs”; besides, some members exist with lower or higher numbers of cysteines, as well as with additional domains [82,83].

Only few vertebrate OBPs (which are structurally different from those of insects) have been studied at the three-dimensional level. The bovine OBP, whose structure was the first to be solved, presented the uncommon phenomenon of domain swapping: the homodimer is stabilised by interactions between the α-helix of one monomer with the β-barrel core of the other [34,35]. The other structures of the same family, namely the pig and the panda [45] OBPs are monomeric, with the α-helical domain closely interacting with the core of the same protein. Other members of the same family, classified with different names only because found in body secretions other than the nasal mucus, but structurally undistinguishable from OBPs, have also been crystallised and their structures solved, in some cases with their bound ligands. These include the urinary proteins of rodents [43], the salivary protein of the pig [36] and the hamster aphrodisin [44]. References to their structures can be found in Table 3, together with those relative to the other two classes of soluble carrier proteins.

Both insect and vertebrate OBPs present very compact structures, allowing reliable models to be made even for other proteins of the same family displaying relatively low sequence homology. This is particularly true for insect OBPs, both because their structure is constrained by the three interlocked disulphide bridges, and thanks to the large number of structures available.

Only three structures of CSPs can be found in the databases, those of the lepidopteran *Mamestra brassicae* [54,55], of the desert locust *Schistocerca gregaria* [56] and the silkmoth *Bombyx mori* [57]. CSPs, that are mostly found in insects, are less rigid and more flexible than OBPs. Such characteristic makes modelling and docking simulation less reliable. To get an idea of how the shape of CSPs can be modified upon ligand binding, it has been reported that the CSP1 of *M. brassicae* can accept three molecules of the large ligand 12-bromododecanol by considerably swelling its binding site [55].

For NPC2 proteins of insects, the forth class of semiochemical binding proteins, only a single structure has been solved [60]. However, NPC2 of vertebrates have been extensively studied as cholesterol and fatty acid carriers and several structures are available [59]. Perhaps it is worth recalling that while in vertebrates a single form of this protein family is expressed in each species, in insects, and more generally in arthropods, NPC2 proteins have undergone a wide process of duplication and differentiation. This phenomenon suggests that such proteins, conserved carriers of lipids in vertebrates, have likely acquired in arthropods the property of binding the diversity of odorants present in the environment [30,32]. From an artificial sensing point of view, the large differentiation of NPC-2 proteins that occurred in the binding pocket to bind a variety of chemical structures, while keeping the external scaffolding almost unaltered, can provide guidelines to design mutants of these proteins to be used in detecting specific ligands of interest.

## 3. Site-Directed Mutagenesis

While all the above discussed characteristics of OBPs and other soluble proteins of chemical communication make them suitable to be incorporated into sensing devices, perhaps their most useful aspect is linked to the possibility of modifying their structures by site-directed mutagenesis to obtain proteins with the desired specificity for odorant molecules. The possibility of designing mutants and predicting their behaviour with a reasonable degree of reliability lies in the many structures so far solved, also in complexes with ligands, and in the detailed knowledge of the architecture of their binding pockets. Moreover, replacing amino acids in specific positions may also serve other purposes, such as that of introducing a fluorescent reporter in the binding site, adding a functional group for immobilization of the protein in a preferential orientation or further stabilising their structure. The following examples can illustrate the potentialities of this technique applied to OBPs.

### 3.1. A Fluorescent Reporter in the pigOBP1

**Figure 2:**
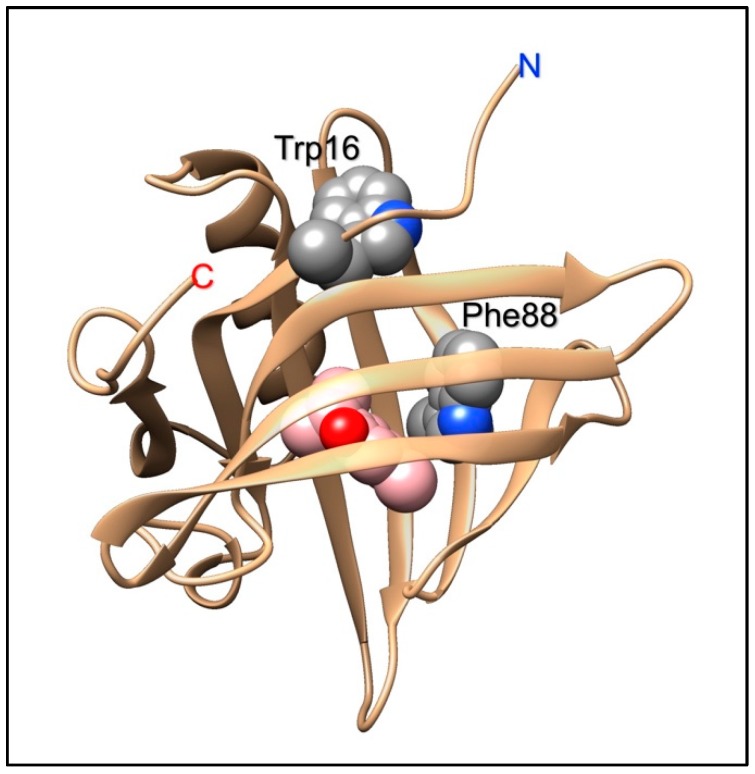
Structure of the pig OBP1. Tryptophan 16 is part of the lipocalin signature and is located outside the binding pocket. In a mutant designed to act as biosensing element for polyaromatic hydrocarbons, phenylalanine 88, which is located inside the binding pocket, was mutated into a second tryptophan residue, that would act as fluorescent reporter to monitor the presence of aromatic ligands in the binding site.

The porcine OBP, like all OBPs of vertebrates, presents a conserved tryptophan, part of the lipocalin signature [40], rather close to the N-terminus and located in the folded protein away from the binding pocket. In order to monitor the binding of aromatic ligands without the use of an external probe, a second tryptophan residue was introduced in the binding pocket by replacing the existing phenylalanine in position 88 [84]. Thanks to the presence of this bicyclic aromatic residue, the mutated protein exhibited higher affinity to a large number of polycyclic aromatic hydrocarbons, as compared to the wild-type. Moreover, the binding of these ligands could be efficiently monitored without the need of external probes, but simply using the intrinsic protein fluorescence of the added tryptophan [84]. Such characteristics make the mutated pig OBP a suitable sensing element to be incorporated into optical devices for monitoring pollutants, such as polyaromatic hydrocarbons in the environment. Interestingly, the replacement of a phenylalanine with a tryptophan produced also the unexpected effect that the modified OBP was able to discriminate between the two enantiomers of carvone [13], unlike the native protein [85]. Figure 2 shows the position of the added tryptophan in the structure of the pigOBP and its proximity to bound ligands.

### 3.2. Narrowing the Specificity of OBPs

**Figure 3:**
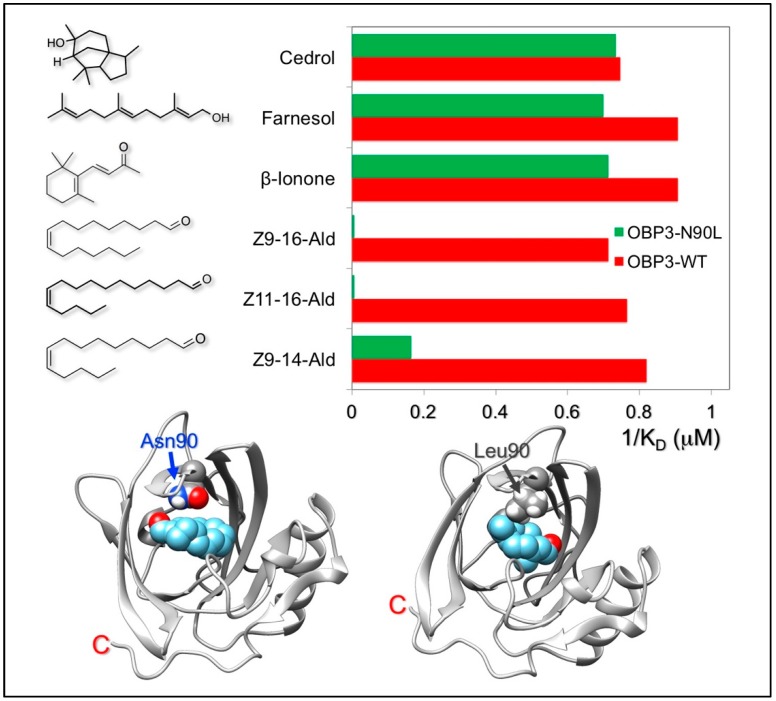
The giant panda OBP3 binds long-chain linear aldehydes as well as terpenoids, including bamboo volatiles. Replacement of asparagine 90 with leucine abolished the affinity of the protein to linear aldehydes while retaining good binding capacity to terpenoids.

Another recent work shows how site-directed mutagenesis can help to obtain more finely tuned sensing elements. The OBP3 of the giant panda binds with good affinity two unrelated classes of chemicals: long-chain linear aldehydes, putative components of the panda pheromone, and some volatile terpenoids of bamboo, components in the flavour of bamboo leaves, the obligated diet of the giant panda [45]. By replacing a single amino acid in the binding pocket (Asn90Leu), the protein lost its affinity to linear aldehydes, while retaining good binding capacity for terpenoids [45]. Figure 3 summarizes the effect of such replacement on the binding properties of the proteins and shows the position of the mutation in the structure of the panda OBP3. Apparently, on the basis of docking simulations, linear aldehydes use Asn90 to establish a hydrogen bond with the protein, while terpenoids bind in the same pocket, but with an opposite orientation. This rationale might explain how the removal of Asn90 drastically reduces the affinity of the protein to linear aldehydes, but does not affect the binding of plant terpenoids.

Another protein in the same animal species, the panda OBP5, shows binding specificity complementary to that of OBP3 [45]. In fact, this protein presents good affinity to linear fatty acids, preferentially to unsaturated members of 16–18 carbon atoms. On the other hand, it does not bind the corresponding aldehydes, nor plant terpenoids, the main ligands of the panda OBP3. A number of proteins with complementary binding specificities, such as OBP3 and OBP5 of the panda, would represent the starting biochemical elements to fabricate a sensor array capable of discriminating different odours on the basis of a combinatorial code approach, borrowing the strategy used by the physiological olfactory system [22].

### 3.3. Modifying the Specificity of OBPs

Another example of site-directed mutagenesis applied to insect OBPs, shows how the tuning of a protein can be switched between different classes of volatiles, using the available literature information.

In Lepidoptera, OBPs can be divided into two different classes, based on their sequences. The first class includes so-called PBPs (pheromone-binding proteins) and GOBPs (general odorant binding proteins). Despite the inappropriate name of the latter, both PBPs and GOBPs are tuned to sex pheromones and share important structural similarities. The remaining OBPs, that can be clearly distinguished from the above on the basis of their sequences, bind plant volatiles, mainly of terpenoid nature. All three PBPs and two GOBPs of the lepidopteran *Plutella xylostella*, a major agricultural pest, present aromatic residues in their binding pockets, able to bind linear long-chain aldehydes (the sex pheromones of this species); OBPs, instead, bear in the same positions other residues, including aliphatic branched amino acids (valine, leucine, isoleucine), suitable to interact with branched terpenoid compounds [86].

**Figure 4:**
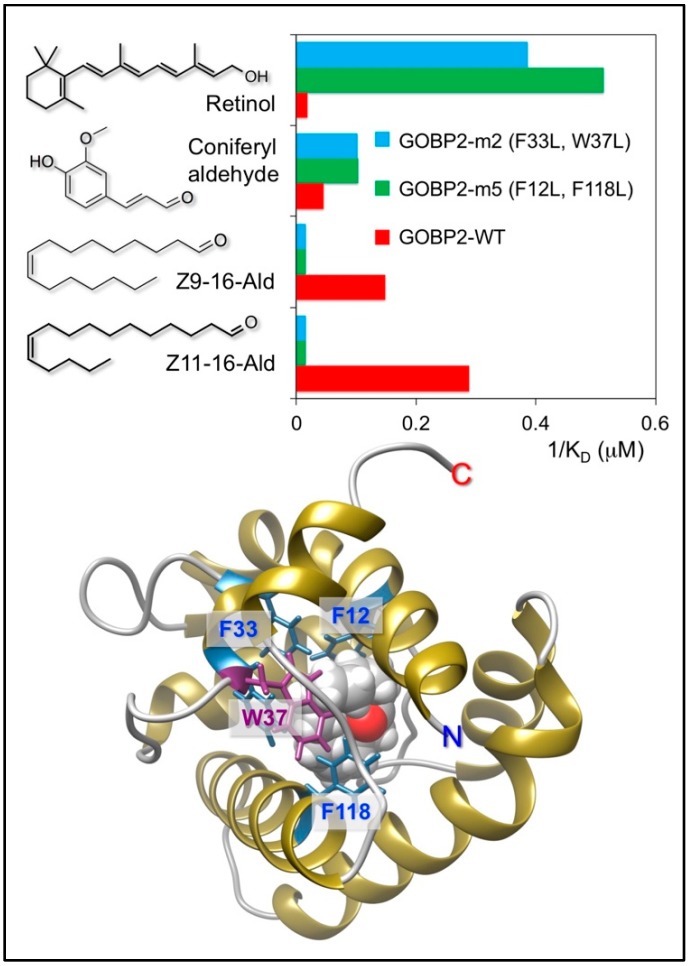
The GOBP2 of the moth *Plutella xylostella* binds with good affinity linear aldehydes, components of the sex pheromone of this species. By replacing two aromatic residues inside the binding pocket with leucine, the specificity of the protein can be switched from linear aldehydes to branched terpenoid compounds.

On this basis of information, the structure of *P. xylostella* GOBP2 was modified by replacing two aromatic residues in its binding pocket with leucine. As a result, the specificity of this protein was switched from linear aldehyde pheromones to branched-chain terpenoids [86]. Figure 4 summarizes the results of this study. This work shows how feasible it could be to completely change the binding properties of an OBP by replacing only one or two residues in the binding pocket, while keeping the scaffolding of the protein virtually intact.

The above examples show how OBPs can be engineered to narrow their selectivity to a small set of ligands. However, we cannot expect to reach the high specificity of enzymes or some receptors. As a matter of fact, in all the cases tested, the mutated proteins retained their affinity to the fluorescent probe, a chemical often structurally different from the ligands of interest. This is due to the flexibility of the protein, but also to the fact that different ligands can sit inside the binding pocket with different orientations. The above cases (both the panda OBP3 and the *Plutella* GOBP2) represent good examples of proteins able to accept ligands of two structurally unrelated chemical classes. In the cases of enzymes, these two set of chemicals would represent the substrates and the competitive inhibitors, respectively.

### 3.4. The Issue of an Aqueous Environment

Despite all these advantages, that indicate OBPs as ideal biosensors for electronic smell detectors, proteins still present some drawbacks, linked to their large size and their hydrophilic nature. Although OBPs are relatively stable, when compared to other proteins, they are far from being rigid and could assume inactive conformation in particularly harsh environments. Certainly, an aqueous medium is strictly required for the protein being functional. Moreover, it might be worth emphasizing the fact that when discussing about stability of OBPs we refer to their unique capability of refolding back into their active conformation after having undergone a denaturing treatment. Instead, permanent denaturing conditions are not compatible with good performance of OBPs.

This represents a major technical issue when immobilising proteins on biochips. On the other hand, odorants by definition are supplied in air, therefore we cannot avoid having an air-water interface when building a protein biosensor, with a series of problems to be solved. First, we should protect the aqueous layer around the protein from evaporation, likely to rapidly occur particularly when the surface of the sensor is kept under a stream of air carrying the odours to be detected. In nature such major problem is solved using different strategies. In the vertebrate nose, a thick layer of mucus, rich in glycoproteins, keeps water molecules tightly bound to the glycan moieties, thus reducing the water vapour pressure and increasing the evaporation time. Nevertheless, evaporation would occur quite rapidly if this mucus layer was not continuously regenerated with new glycoproteins and water supplied by the epithelial cells [87,88].

Insects and other arthropods have adopted a different solution. The sensing elements, the olfactory neurons with their olfactory receptors, are bathed in a special lymph and encapsulated into sensilla within a hard cuticle wall. Tiny pores on the cuticle allow the entrance of odorants, but prevent the exit of water due to its high surface tension. Even in such cases, however, the sensillar lymph is constantly regenerated [89,90].

Although it is technically feasible to put a hydrogel at the interface between the external gaseous environment and the liquid phase in which the detecting proteins are kept, this would introduce a slow step in the biosensor responses, as the odorants, by nature generally hydrophobic, have to cross an aqueous layer, where their low solubility would greatly slow down diffusion towards the biosensing elements.

### 3.5. Recovery of Biosensor Functionality

More serious difficulties are related to regeneration of biosensors in times compatible with a continuous monitoring of the environment. The first problem is again the presence of an aqueous interface where the reduced diffusion rate of odorants slows down their release back into the environment. In biological systems, such as the mammalian nose or the antenna of an insect, active mechanisms of washing and regenerating olfactory receptors are adopted with a continuous flow of nasal mucus or the fast replacement of sensillar lymph [87,89].

Another problem is related to the performance of the proteins so far adopted in biosensors for odours. Both OBPs of vertebrates and those of insects exhibit dissociation kinetics rather slow, of the order of minutes for insect OBPs and hours for the mammalian proteins [14,65,91]. With the latter proteins, in particular with the bovine OBP it has been observed that there is a gated entrance of the ligand. The benzene ring of a phenylalanine residue acts as a door opening for letting the odorant inside the binding pocket and then returning to a closed position, thus greatly slowing the process of dissociation [34].

This feature, while a major problem when monitoring in real time is desired, could represent an advantage if the protein is used in a sort of dosimeter, accumulating specific odorants of interest, that may be generated from degrading foods or in unhealthy environments.

As a consequence, the biosensors described so far are mostly used to detect ligands in solution and are characterized by times of response and recovery very long as compared to those of biological olfactory systems. Such devices can still be valuable when used as analytical instruments in a laboratory, but are not yet suitable for real time monitoring of environmental odours. Besides, we should emphasize the fact that the biosensors so far developed provide a “proof of concept” rather than devices designed for specific technological use. In this respect, it is unlikely that sensors tuned to insect pheromones might find industrial applications; however, they provide a unique system where the components (pheromones and binding proteins) are know and well characterised, to test the performance of a biosensor and compare its responses with those of the biological system.

## 4. Conclusions

Based on the properties described above, OBPs and other soluble proteins involved as odorant carriers in chemical communication, can be considered as the best candidates currently available to be used as detecting elements in biosensors for odours.

As proteins, they possess the required specificity to discriminate among the large variety of odours that our nose can recognise. At the same time, they are exceptionally stable compared to other proteins, a characteristic probably related to the fact that they work at the interface between the sensory organs and the external environment.

However, the most promising characteristic of these proteins is the possibility of modifying their ligand-binding specificity by simple techniques of site-specific mutagenesis. Such opportunity is made feasible thanks to the wide and detailed information on the structures of many OBPs, and is documented by several successful projects.

In particular, some results have shown how the properties of the binding cavity can be modified while keeping the scaffolding of the protein unaltered. This fact may suggest that in the future we might be able to design and synthesise new proteins with the desired binding specificity, endowed at the same time with improved characteristic of stability, specific optical properties and suitability to be immobilised onto electronic chips, among other physical properties.

## Figures and Tables

**Table 1:** Soluble proteins used for chemical communication across evolution. The small number of CSPs in Crustacea (1–3) probably does not support a role in chemical communication. NPC2 proteins occur in all phyla, but only in invertebrates their number and diversity indicate a function in chemical communication.

	VerOBPs	InsOBPs	CSPs	NPC2	OBP-Like
Hexapoda		●	●	●	
Crustacea			●	●	
Chelicerata				●	●
Myriapoda					●
Amphibia	●				
Mammalia	●				

**Table 2:** Structures of insect OBPs. Accession numbers are: MMDB; PDB.

*Species*	*Protein*	*Acc. n. (apo)*	*Acc. n. (olo)*
**Diptera**			
*Aedes aegypti*	OBP1	78,632, 3K1E	
*Aedes aegypti*	Aed7	69,416, 3DXL 69,417, 3DY9	69,419, 3DZT 69,418, 3DYE
*Amyelois transitella*	PBP1	79,681, 2KPH (NMR, pH 4.5)	108,208, 4INX 108,207, 4INW
*Anopheles gambiae*	OBP7	94,463, 3R1P 94,462, 3R1O	94,464, 3R1V
*Anopheles gambiae*	OBP20	103,980, 3VB1 104,060, 4F7F	103,979, 3V2L
*Anopheles gambiae*	OBP1		91,180, 3N7H 142,574, 5EL2
*Anopheles gambiae*	OBP47	90,818, 3PM2	
*Anopheles gambiae*	OBP48	113,919, 4KYN	113,894, 4IJ7
*Anopheles gambiae*	D7r4	59,449, 2QEV	59,448, 2QEO 59,445, 2QEH 59,443, 2QEB 59,316, 2PQL
*Culex quinquefasciatus*	OBP		85,954, 3OGN
*Drosophila melanogaster*	LUSH	32,732, 1T14 23,924, 1OOI (pH 6.5)	23,923, 1OOH 23,922, 1OOG 23,921, 1OOF 46,137, 2GTE
*Drosophila melanogaster*	LUSH T57A		62,295, 3B88 62,294, 3B87
*Drosophila melanogaster*	LUSH T57S		62,293, 3B86
*Drosophila melanogaster*	LUSH S52A		62,291, 3B6X
*Drosophila melanogaster*	LUSH D118A	64,745, 2QDI	
*Drosophila melanogaster*	LUSH S52A		62,292, 3B7A
*Lutzomyia longipalpis*	SALO	141,432, 5KX4	
*Phlebotomus duboscqi*	CPI	113,901, 4JD9	
**Lepidoptera**			
*Antheraea polyphemus*	PBP1	26,871, 1QWV 60,086, 2JPO (pH 4.5, NMR) 35,432, 1TWO (NMR, low pH)	
*Bombyx mori*	PBP1	37,387, 2FJY 21,174, 1LS8 18,041, 1GM0 15,475, 1DQE 35,031, 1XFR	45,969, 2P70 4597, 2P71
*Bombyx mori*	GOBP2	75,865, 2WC5 75,869, 2WCK	75,871, 2WCM 75,870, 2WCL 75,868, 2WCJ 75,867, 2WCH 75,866, 2WC6
**Hymenoptera**			
*Apis mellifera*	ASP1	61,456, 3BJH 64,870, 3CDN 61,126, 2H8V	78,407, 3FE9 64,850, 3BFH 64,849, 3BFB
*Apis mellifera*	ASP2		34,972, 1TUJ (NMR)
*Apis mellifera*	OBP5	98,491, 3R72	
*Apis mellifera*	OBP14	95,366, 3S0F 95,362, 3S0A	95,365, 3S0E 95,364, 3S0D 95,363, 3S0B 95,361, 3RZS
*Apis mellifera*	OBP14 Q44C, H97C	95,367, 3S0G	
*Apis mellifera*	OBP14 D35N	72,884, 3D78	
*Apis mellifera*	OBP14 D35A	72,880, 3D74 72,879, 3D73	
**Coleoptera**			
*Tenebrio molitor*	THP12	11,597, 1C3Z 11,596, 1C3Y	
**Orthoptera**			
*Locusta migratoria*	OBP1	125,780, 4PT1	
**Blattodea**			
*Rhyparobia maderae*	PBP1	24,088, 1ORG	24,093, 1OW4 24,102, 1P28

**Table 3:** Structures of mammals OBPs, and insect CSPs and NPC2. Accession numbers are: MMDB; PDB.

*Species*	*Protein*	*Acc. n. (apo)*	*Acc. n. (olo)*
**Mammals OBPs**			
*Bos taurus*	OBP1	50,597, 1OBP	19,732, 1G85 6240, 1PBO 18,101, 1HN2 24,552, 1GT5 72,179, 1GT1 71,060, 1GT3 24,551, 1GT4
*Bos taurus*	OBP1 mut	63,212, 2HLV	
*Sus scrofa*	OBP1	71,515, 1A3Y	15,148, 1DZK 15,150, 1DZP 15,147, 1DZJ 15,149, 1DZM 15,153, 1E06
*Sus scrofa*	SAL	19393, 1GM6	
*Ailuropoda melanoleuca*	OBP3	155,697, 5NGH	
*Mus musculus*	MUP9	56,909, 1MUP 13,488, 1DF3	
*Rattus norvegicus*	OBP1	72,769, 3FIQ	
*Rattus norvegicus*	α-2u-globulin		11,004, 2A2G 11,005, 2A2U
*Mesocricetus aureus*	Aphrodisin	71,930, 1E5P	
**Insect CSPs**			
*Mamestra brassicae*	CSP2 (A6)	21,132, 1KX9 21,100, 1K19	21,132, 1KX9
*Schistocerca gragaria*	CSP1 (CSP4)	41,578, 2GVS	
*Bombyx mori*	CSP1	60,768, 2JNT	
**Insect NPC2**			
*Camponotus japonicus*	NPC2	117,138, 3WEA	117,139, 3WEB
